# A motor neuron disease mouse model reveals a non-canonical profile of senescence biomarkers

**DOI:** 10.1242/dmm.049059

**Published:** 2022-08-29

**Authors:** Pascual Torres, Carlos Anerillas, Omar Ramírez-Núñez, Anna Fernàndez, Mario Encinas, Mònica Povedano, Pol Andrés-Benito, Isidre Ferrer, Victòria Ayala, Reinald Pamplona, Manuel Portero-Otín

**Affiliations:** 1 Metabolic Pathophysiology Research Group, Department of Experimental Medicine, University of Lleida-IRBLleida, 25196 Lleida, Spain; 2 Oncogenic Signalling and Development, Department of Experimental Medicine, University of Lleida-IRBLleida, 25196 Lleida, Spain; 3 Functional Unit of Amyotrophic Lateral Sclerosis (UFELA), Service of Neurology, Bellvitge University Hospital, 08907 Hospitalet de Llobregat, Barcelona, Spain; 4 Department of Pathology and Experimental Therapeutics, University of Barcelona, 08907 Hospitalet de Llobregat, Barcelona, Spain; 5 Biomedical Network Research Center on Neurodegenerative Diseases (CIBERNED), Institute Carlos III, 08907 Hospitalet de Llobregat, Barcelona, Spain

**Keywords:** Amyotrophic lateral sclerosis, Navitoclax, Senolytic, Neuroinflammation, Therapy, Cell cycle, Cryptic exon

## Abstract

To evaluate senescence mechanisms, including senescence-associated secretory phenotype (SASP), in the motor neuron disease model hSOD1-G93A, we quantified the expression of *p16* and *p21* and senescence-associated β-galactosidase (SA-β-gal) in nervous tissue. As SASP markers, we measured the mRNA levels of *Il1a*, *Il6*, *Ifna* and *Ifnb*. Furthermore, we explored whether an alteration of alternative splicing is associated with senescence by measuring the *Adipor2* cryptic exon inclusion levels, a specific splicing variant repressed by TAR DNA-binding protein (TDP-43; encoded by *Tardbp*). Transgenic mice showed an atypical senescence profile with high *p16* and *p21* mRNA and protein in glia, without the canonical increase in SA-β-gal activity. Consistent with SASP, there was an increase in *Il1a* and *Il6* expression, associated with increased TNF-R and M-CSF protein levels, with females being partially protected. TDP-43 splicing activity was compromised in this model, and the senolytic drug Navitoclax did not alter the disease progression. This lack of effect was reproduced *in vitro*, in contrast to dasatinib and quercetin, which diminished *p16* and *p21*. Our findings show a non-canonical profile of senescence biomarkers in the model hSOD1-G93A.

## Abstract (precis)

**Summary:** The hSOD1-G93A mouse, a motor neuron disease model, shows an atypical profile of molecular traits of cell senescence in the lumbar spinal cord, with selective sensitivity to senolytic treatments.

## INTRODUCTION

Aging is a major risk factor for developing amyotrophic lateral sclerosis (ALS) (Niccoli et al., 2017). ALS is a neurodegenerative disease characterized by losing motor neurons with an unfavorable outcome (<5% survival at 5 years after diagnosis). Cellular senescence was first described by Hayflick in the 1960s as a limitation on division of normal cells *in vitro* (Hayflick and Moorhead, 1961). The cellular mechanisms behind this phenomenon were later described, with an important role for cell cycle inhibitors, highlighting p16-INK4A as the major contributor (Serrano et al., 1997). Another hallmark of senescent cells is the increase in senescence-associated β-galactosidase (SA-β-gal), which is associated with an increase in lysosomal biogenesis (Kurz et al., 2000). Cellular senescence has been described as a barrier against oncogenesis, with a tradeoff where these cells can develop a pro-inflammatory status known as senescence-associated secretory phenotype (SASP). This process reflects an attempt to induce tissue repair in which senescent cells, usually accumulating DNA damage, can stimulate its clearance by the immune system. Regarding neurodegenerative diseases, several groups have independently demonstrated the presence of senescent glial cells and SASP in the central nervous system (CNS).

Another process related to aging is the change in alternative splicing (AS), a conserved mechanism that increases the complexity of the proteome. TAR DNA-binding protein (TDP-43; encoded by *TARDBP*) regulates many AS events in a complex way (Tollervey et al., 2011). Several pieces of evidence support the role of TDP-43 pathology in age-related neurodegenerative processes and physiological aging (McAleese et al., 2017). Most AS events regulated by TDP-43 involve the repression of a set of non-conserved (cryptic) exons that are abnormally incorporated into mRNA in ALS (Ling et al., 2015). In this line, we previously quantified the rate of inclusion of cryptic exons in nervous tissue from ALS donors and cellular models and found a positive correlation with age at death (Torres et al., 2018).

To study the potential involvement of senescence-associated phenomena in ALS, we explored the profile of senescence biomarkers, including the expression and protein levels of cell cycle modulators linked to mitosis arrest (p16 and p21). We also quantified soluble factors associated with stress-induced senescence secretory profile in motor neuron degeneration in transgenic models, linked to overexpression of mutated proteins. To further strengthen the potential translational power of our findings, we also evaluated whether known senolytic strategies can modify the phenotype of the models and compared these findings with an *in vitro* model of fibroblast senescence. The results demonstrate that neuroinflammation phenomena associated with SOD1-G93A overexpression are intertwined with senescence-associated secretory profiles. Not all senolytic approaches [i.e. Bcl-2/Bcl-XL (also known as BCL2L1)/Bcl-w (also known as BCL2L2)] and antioxidant ones have the same outcome, stressing adequate preclinical modeling of the disease.

## RESULTS

**Fig. 1.**
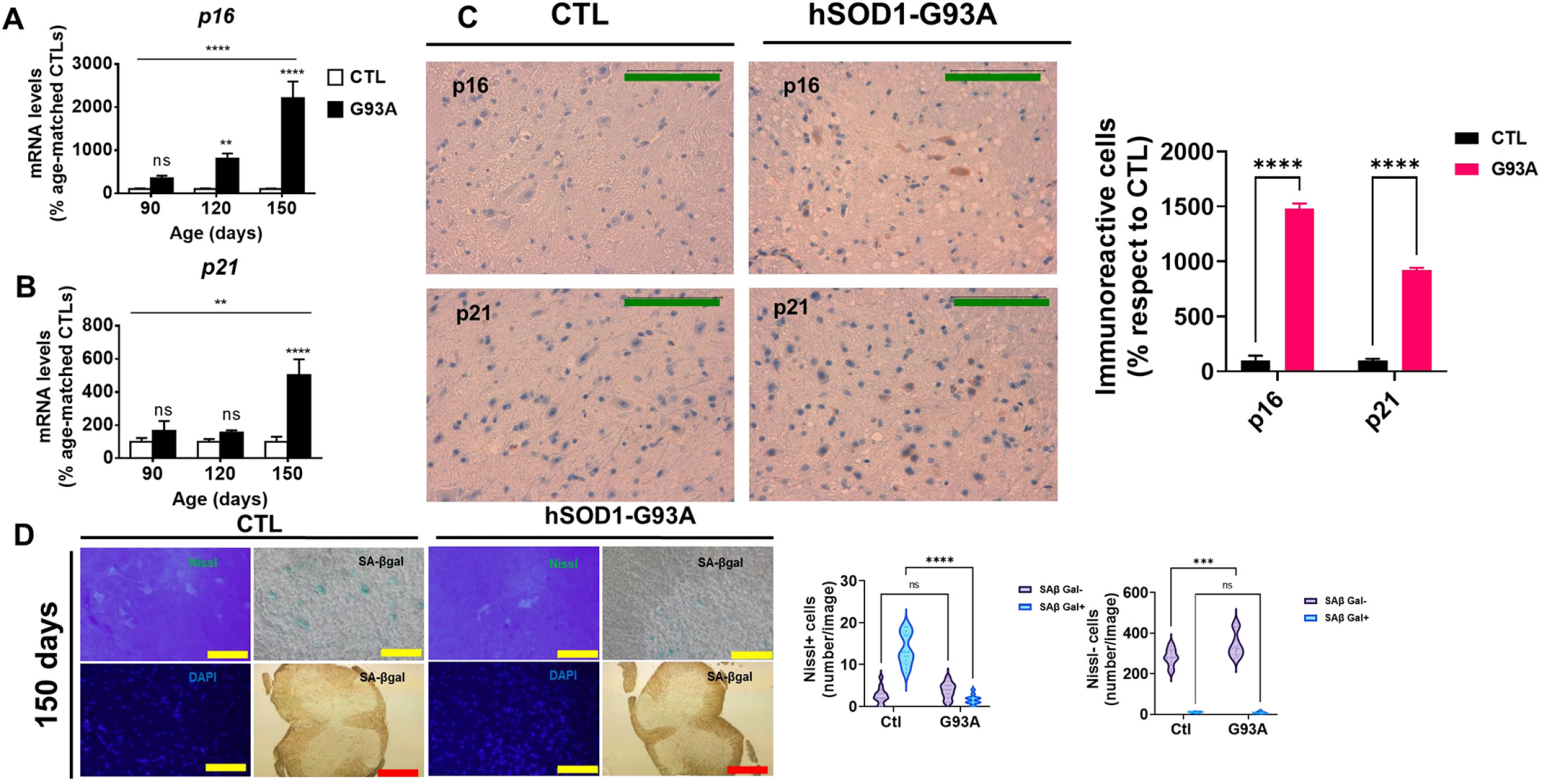
**Senescence markers in spinal cord increase during amyotrophic lateral sclerosis (ALS) progression.** (A,B) Effect of age on *p16* (A) and *p21* (B) mRNA expression. (C) p16 and p21 cytoplasmic staining, quantified in the right bar graph. (D) Nissl^+^ staining of the ventral horn of the spinal cord and senescence-associated β-galactosidase (SA-β-gal) activity in the lumbar spinal cord at 150 days, both quantified in the violin plots shown on the right. p16 and p21 expression data are expressed as mean±s.e.m. ns, *P*>0.05; **P<0.01; ***P<0.001; ****P<0.0001 unpaired two-tailed Student's *t*-test or two-way ANOVA). Green scale bars: 100 µm; yellow scale bars: 500 µm; red scale bars: 2500 µm. *n*=4 from each genotype and age. Ctl, control.

To clarify whether senescence-associated phenomena and TDP-43 dysfunction could be implicated in ALS, we measured the abovementioned variables in the familial ALS transgenic mouse model hSOD1-G93A at different disease stages. The senescence markers *p16* (also known as *Cdkn2a*) and *p21* (also known as *Cdkn1a*), specific biomarkers of senescent cells (Coppé et al., 2010), were analyzed in lumbar spinal cord (LSC). In control mice, we did not observe an age-related increase in senescence biomarkers such as *p16*, *p21*, *Il1a*, *Il6* or exon retention percentage. This lack of variation is in line with the fact that changes between 90 and 150 days of age cannot be considered aging in this strain, with a medium survival ranging between 27 and 29 months (Graber et al., 2015). We proposed that G93A overexpression, most probably via secretion of neuroinflammatory mediators, might act like a pro-senescence stimulus in the CNS. Using two different techniques [immunohistochemistry (IHC) and immunofluorescence (IF)], we characterized the tissue distribution pattern of p16. The results from reverse transcription quantitative PCR (RT-qPCR) showed that the expression of *p16* mRNA was progressively increased during disease evolution (Fig. 1A), whereas *p21* mRNA levels were only higher at the end stage (Fig. 1B). p16 and p21 exhibited a predominantly cytoplasmic pattern (Fig. 1C; detailed in Fig. S1), in contrast to recent results from an ALS rat model that showed mainly nuclear expression (Trias et al., 2019). To define the potential senescence incidence in this model further, we also analyzed another senescence canonical biomarker, SA-β-gal activity. Although increases in SA-β-gal activity are less easily demonstrable in mouse cells than in human cells (Raffaele et al., 2020), we have shown that there are several neurons (and non-neuronal cells) exhibiting this activity, which is in line with findings of other researchers (Itahana et al., 2007). The main cellular populations expressing SA-β-gal in ventral LSC are the motor neuron cells (Nissl^+^ cells in the ventral horn, with a cellular size compatible with that of motor neurons). Neurons of other LSC locations and the vast majority of Nissl^−^ cells do not show SA-β-gal activity (Fig. 1D; Fig. S2). Interestingly, SA-β-gal activity was reduced during disease progression in motor neurons and a small fraction of Nissl^−^ cells (compatible with glia).

**Fig. 2.**
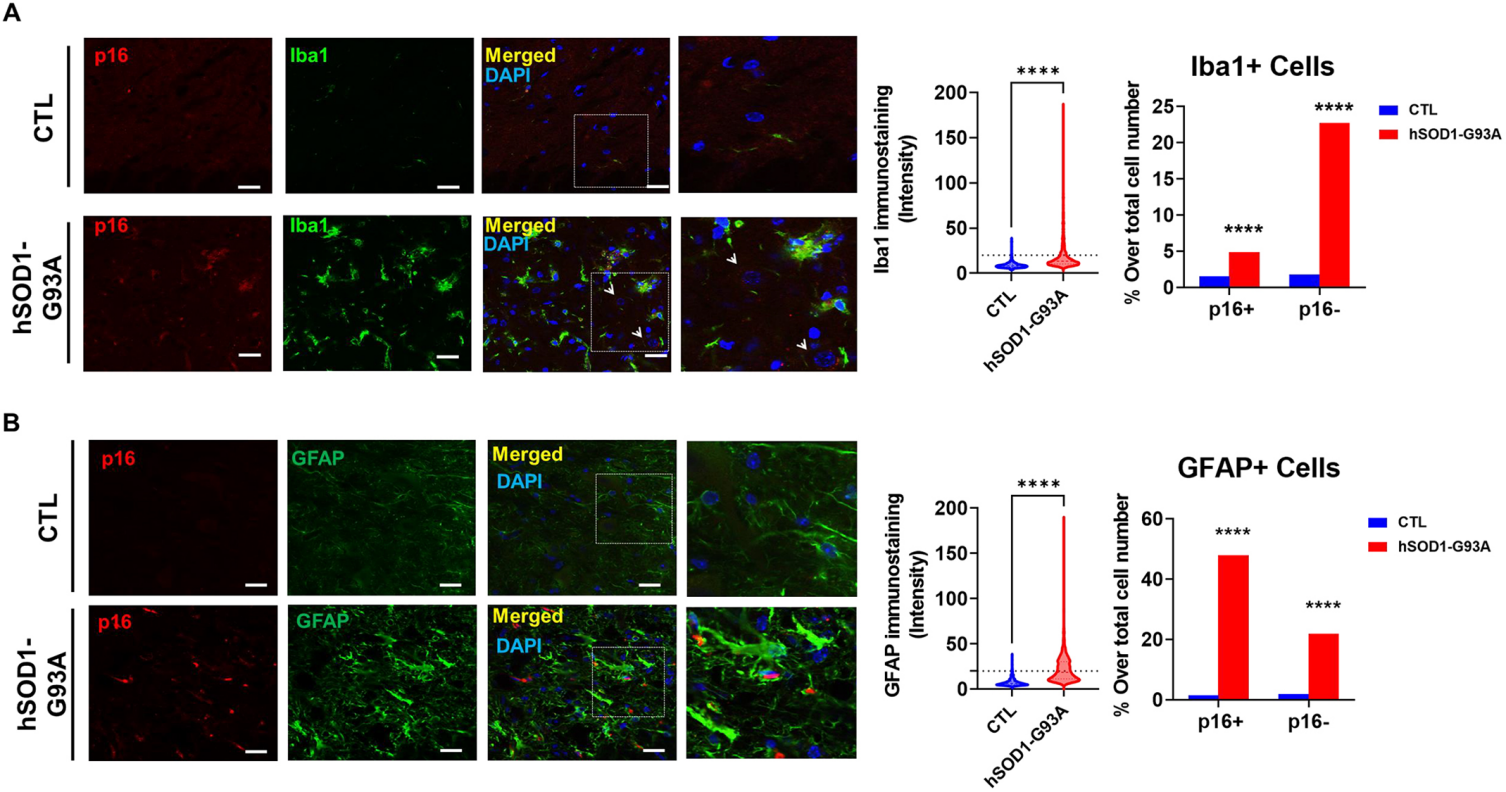
**p16 immunofluorescence is present in microglia and astrocytes.** (A,B) Cytoplasmic p16^+^ microglia (Iba1^+^) (A) and astrocytes (GFAP^+^) (B) in the lumbar spinal cord (LSC) of transgenic mice and controls. The rightmost images show amplifications of the areas within the boxes. Right panels show quantitative analyses of these images, with violin plots showing the mean cellular intensity of Iba1 (A) and GFAP (B), and horizontal dotted lines in violin plots indicating intensities used for selecting Iba1^+^ and GFAP^+^ cells. Bar graphs indicate the proportion of p16^+^ and p16^−^ cells across this population. ****P<0.0001 (unpaired two-tailed Student's *t*-test in violin plots, or Chi-square test of proportions in bar graphs). Scale bars: 20 µm. Representative images from independent experiments (*n*=10 slices from at least three different animals from each genotype) are shown. Arrowheads indicate nuclei of motor neurons with p16 immunoreactivity.

Confocal IF confirmed non-nuclear p16 staining (Fig. 2A,B, rightmost column). hSOD1-G93A overexpression led to an increase in p16 immunoreactivity, with most p16^+^ cells being microglia (Iba1^+^ cells) (Fig. 2A) or astroglia (GFAP^+^ cells) (Fig. 2B). Quantitative analyses of immunoreactivity distribution suggested than the percentage of p16^+^ cells is higher in astrocytes than in microglia (Fig. 2).

**Fig. 3.**
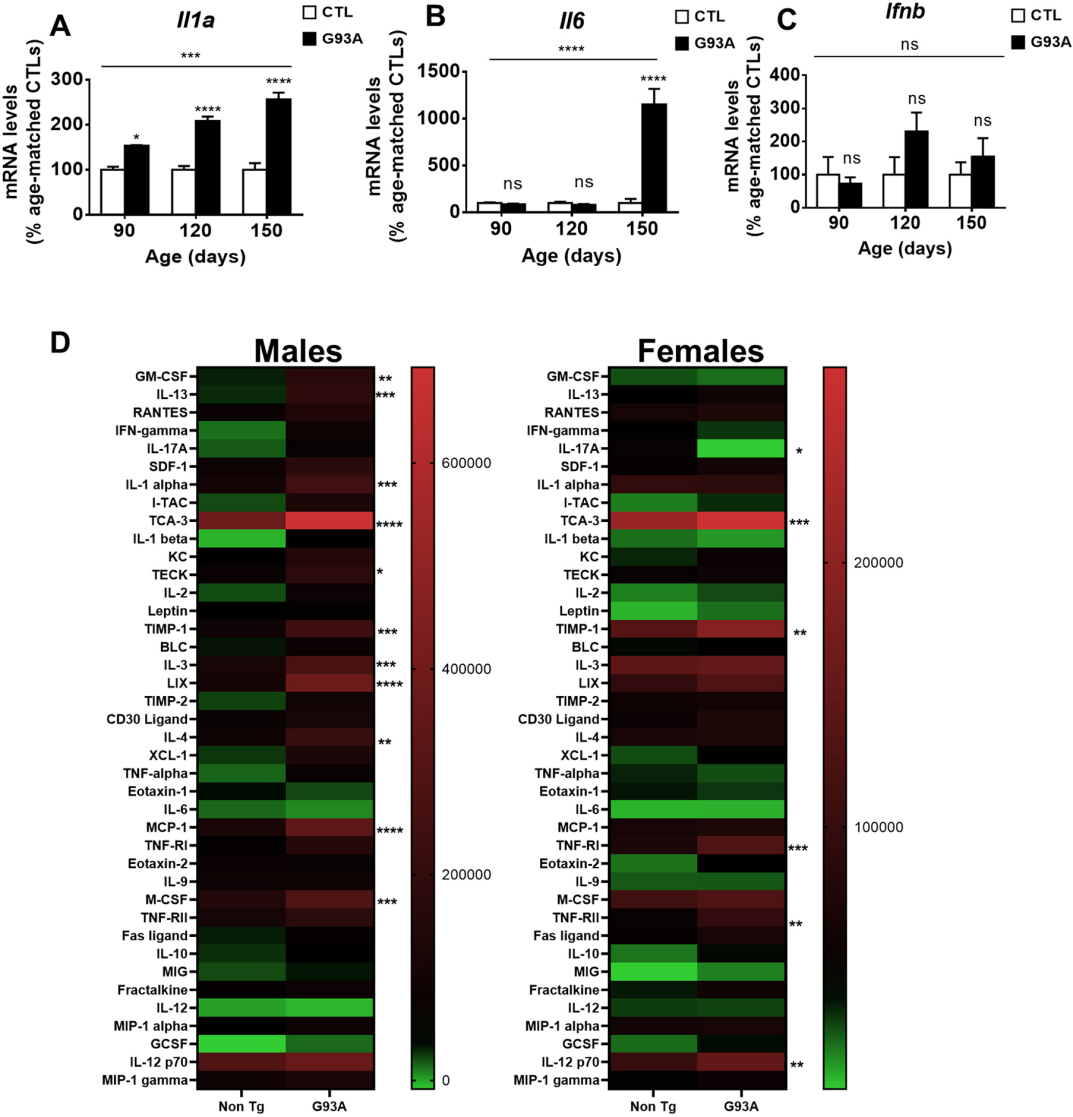
**Senescence-associated secretory phenotype (SASP) markers are increased in LSC from transgenic hSOD1-G93A mice in a sex-dependent manner.** (A-C) *Il1a* (A), *Il6* (B) and *Ifnb* (C) mRNA expression in LSC. (D) Heat maps showing median levels (scale present right of the heatmaps) of inflammatory proteins in LSC in G93A mice at the presymptomatic stage (90 days). *Il1a*, *Il6* and *Ifnb* mRNA levels are expressed as mean±s.e.m. ns, *P*>0.05; *P<0.05; ***P<0.001; ****P<0.0001 (Fisher’s least significant difference in post hoc test after one-way ANOVA). In A-C, *n*=4 from each genotype and age; in D, *n*=2 from each genotype and sex.

Regarding the SASP, we analyzed the expression of *Ifna* and *Ifnb* (also known as *Ifnb1*) (corresponding to type-I IFN response) (Nakao et al., 2020) as they are postulated as late-senescence markers and could help determine senescence progression in the LSC of this model. The expression of *Ifna* was not detected in any of the analyzed samples. We observed a different expression pattern between *Il1a* (Fig. 3A) and *Il6* (Fig. 3B). *Ifnb* expression (Fig. 3C) was not altered over the experimental period, which could indicate that senescence in this model does not evolve in a late phase. To shed further light on the role of neuroinflammation and SASP, we evaluated an array of inflammatory proteins in the spinal cord in a small subset of samples. Sex strongly influenced the effect of G93A overexpression on the levels of these proteins, with females being partially protected against it (Fig. 3D). Thus, although in male mice, G93A overexpression explained 11.8% of the total variance (*P*<0.0001 in two-way ANOVA), it only explained 1.7% of the total variance in female mice (*P*<0.0001 in two-way ANOVA). Regarding specific proteins, TCA-3 (also known as CCL1) was the only differential protein shared by males and females (*P*<0.001 and *P*<0.05 in Bonferroni post hoc after two-way ANOVA). Using less stringent statistical analyses (Fig. S3), four different proteins were commonly changed in male and female mice [TCA-3, TIMP-I, TNF-RI (also known as TNFRSF1A) and TNF-RII (also known as TNFRSF1B)]. We also analyzed the published RNA-sequencing (RNASeq) datasets of G93A mouse-derived cells for the measured markers of senescence quantified here (Chiu et al., 2013; Liu et al., 2020; Phatnani et al., 2013). The results (Fig. S3) indicate that, in line with our findings, microglial levels of *Il1a*, *p21* and *Csf1* were increased by G93A overexpression (Chiu et al., 2013). Furthermore, increased astrocyte *p21* expression was detected by independent researchers (Liu et al., 2020).

**Fig. 4.**
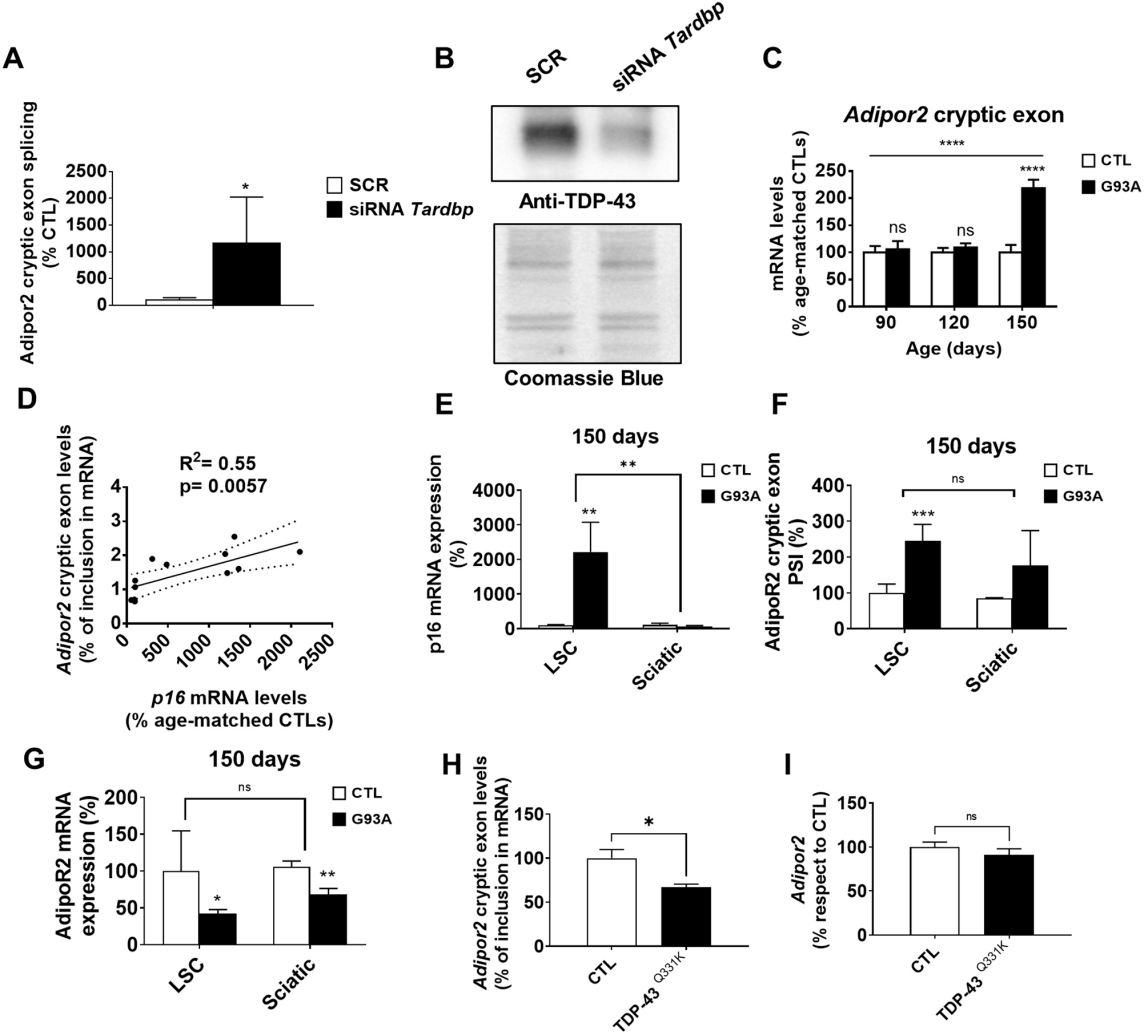
**Cell senescence could impair TDP-43 function in G93A mice.** (A,B) RT-qPCR of *Adipor2* cryptic exon (A) and western blot analyses of TDP-43 in *Tardbp*-silenced 3T3 cells in comparison to scrambled siRNA (SCR) (B). (C) Cryptic exon in *Adipor2* mRNA in LSC from 150-day-old G93A transgenic mice. (D) The inclusion ratio of *Adipor2* cryptic exon positively correlated with *p16* expression in LSC. (E-G) *p16* expression (E), *Adipor2* mRNA cryptic exons (F) and total *Adipor2* mRNA (G) in sciatic nerves and LSC from 150-day-old mice. PSI, percent spliced in or cryptic exon-inclusion ratio. (H,I) *Adipor2* cryptic exon (H) and total mRNA (I) in LSC from TARDBP^Q331K^-overexpressing 150-day-old mice. *p16* and cryptic *Adipor2* expression are expressed as mean±s.e.m. ns, *P*>0.05; *P<0.05; **P<0.01; ***P<0.001; ****P<0.0001. *n*=4 from each genotype and age. In A and B, data shown represent three independent experiments.

Regarding TDP-43 splicing function, in mice, it controls the inclusion of *Adipor2* mRNA (Fig. 4A,B). In line with the loss of TDP-43 function in this model, cryptic exon inclusion in *Adipor2* mRNA was higher in the LSC in end-stage mice (Fig. 4C) and positively correlated with *p16* expression (Fig. 4D). Increased p16 seemed to be restricted to the CNS, as the sciatic nerve did not show these changes (Fig. 4E), in contrast to *Adipor2* cryptic exon inclusion, which was also increased in the sciatic nerve (Fig. 4F). Noteworthily, increased *Adipor2* cryptic exon was associated with loss of *Adipor*2 mRNA levels, suggesting increased nonsense-mediated decay in both locations (Fig. 4G). To evaluate whether enhanced TDP-43 leads to diminished *Adipor2* cryptic exon inclusion *in vivo*, we analyzed *Adipor2* splicing in the TARDBP^Q331K^ transgenic mouse, which showed a gain of splicing function (Fratta et al., 2018). At 150 days, when these mice did not show motor neuron phenotype, *Adipor2* cryptic splicing was reduced in LSC (Fig. 4H), with total *Adipor2* mRNA remaining unaltered (Fig. 4I).

**Fig. 5.**
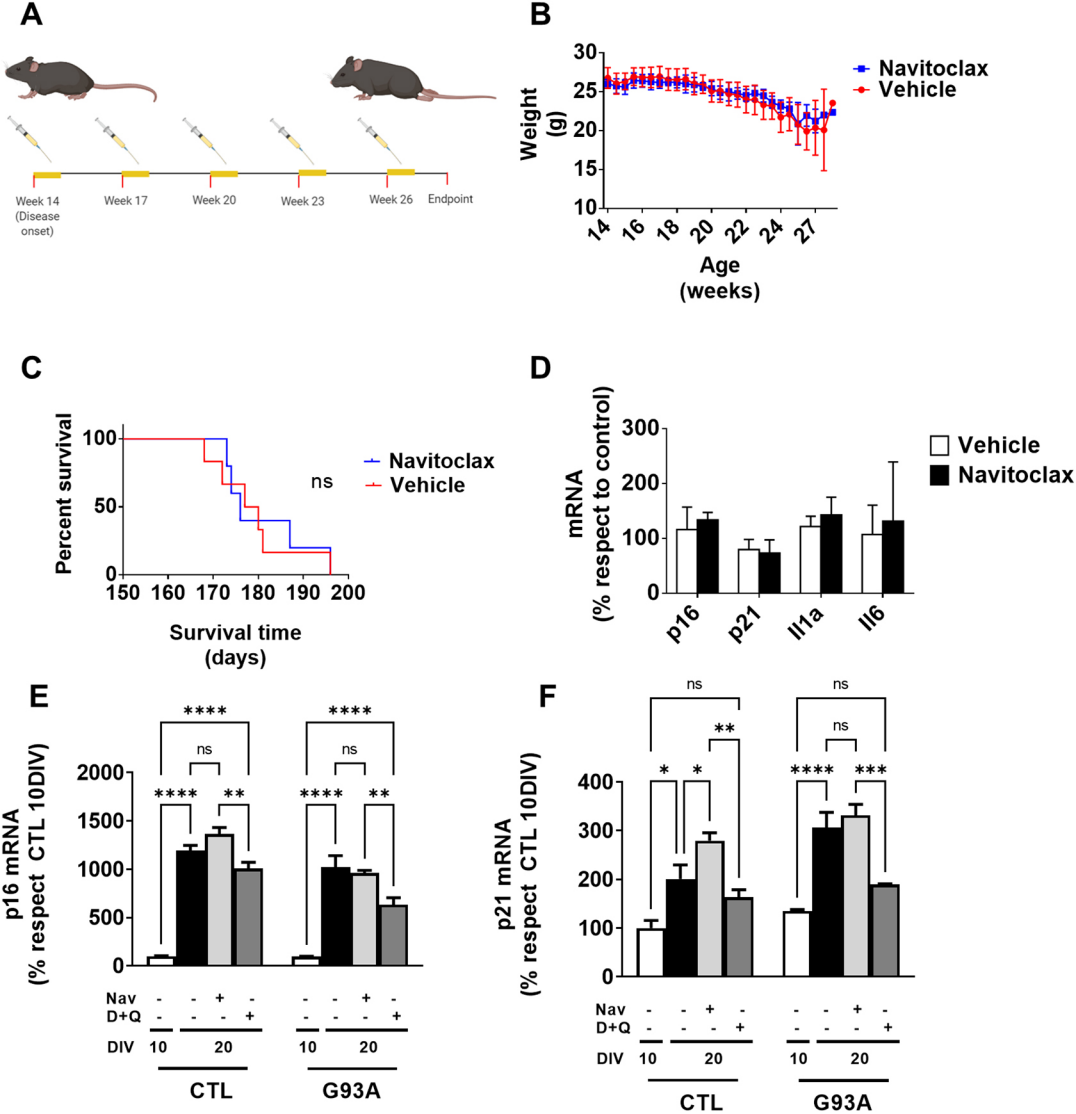
**Navitoclax treatment does not slow disease progression in G93A mice.** (A) A chronic treatment *in vivo* was established with five consecutive doses followed by 2 weeks of resting. (B-D) Effect of chronic Navitoclax treatment on weight loss (B), survival time (C) and expression of SASP genes (D). (E,F) Effect of alternative senolytic regime [dasatinib and quercetin (D+Q)] in comparison to Navitoclax (Nav) on the expression levels of *p16* (E) and *p21* (F) mRNA *in vitro*. DIV, days *in vitro*. In A-D, *n*=5 from each genotype. In E and F, data shown represent three different experiments. Data are expressed as mean±s.e.m. ns, *P*>0.05; *P<0.05; **P<0.01; ***P<0.001; ****P<0.0001 (Bonferroni post hoc analyses after two-way ANOVA).

We wanted to explore the potential benefits of senolytic treatments due to the higher expression of senescence-related genes in the G93A model. We performed Navitoclax treatment following the protocol described for an Alzheimer's disease mouse model (Bussian et al., 2018). The treatment was initiated at 90 days and finished at the endpoint (Fig. 5A). We estimated the disease progression by weight loss. Navitoclax treatment did not prevent weight loss or prolong survival (Fig. 5B,C). Finally, we quantified senescence and SASP genes in the LSC. None of the analyzed genes showed statistically significant differences (Fig. 5D). Because Navitoclax treatment, which focused on Bcl-2/Bcl-XL/Bcl-w inhibition, did not significantly affect survival, we tried a different senolytic regime in a fibroblast cell culture. Previous data support using a multitarget strategy, such as dasatinib and quercetin combination, as a senolytic approach (Hickson et al., 2019). The results (Fig. 5E,F) showed that G93A expression significantly impacts both *p16* (6.75% of variance explained by genotype, *P*<0.001, two-way ANOVA) and *p21* (10.8% of variance, *P*<0.0001, two-way ANOVA) mRNA levels. Interestingly, although Navitoclax had a negligible effect on the concentration of these mRNAs, the dasatinib–quercetin combination significantly decreased p16 and p21 mRNA levels compared to vehicle (*P*<0.0005 and *P*<0.0001, respectively).

## DISCUSSION

The expression profiles of cell senescence-linked kinases (*p16* and *p21*) suggest that senescence-associated markers and SASP are related to disease evolution in the G93A model. *p16* expression is highly expressed before the symptomatology in these mice, similarly to p16^+^ microglia in LSC from transgenic rats (Trias et al., 2019). Both findings suggest a role for p16 in disease initiation and progression, as *p16* mRNA is increased up to an early symptomatic stage (120 days), whereas *p21* mRNA only increases later. *p21* mRNA upregulation (related to reversible cell cycle arrest or quiescence) might derive from the late-onset activation of p53 and the DNA damage response pathway, as shown for senescent microglia (Stojiljkovic et al., 2019). Although p21 does not get upregulated in all models, it has a clear role in senescence, both in a p53- dependent and -independent fashion (Shtutman et al., 2017). Usually, senescence – specially for human cells – implies stability in cell cycle arrest. In contrast, mouse cells may not actually undergo bona fide senescence, but rather a profile of stress-induced cessation of growth and buildup of a secretory profile associated with senescence. The pattern of p16 and p21 expression reported here may reflect divergent processes sharing these biomarkers (Sharpless and Sherr, 2015). This is the case with macrophage polarization, in which p16 expression and SA-β-gal activity are physiological, reversible and not associated with cellular senescence (Hall et al., 2017).

Regarding the subcellular location of p16 and p21, cytoplasmic p16 can regulate cell migration like cyclin D1 does (Chen et al., 2013). Cytoplasmic p21 inhibits the ROCK/LIMK/Cofilin pathway through MAPK signaling, inducing cytoskeleton remodeling (Tanaka et al., 2002). Indeed, cytoskeleton regulators like RAC1 and CDC42 are implicated in ALS progression and neuroinflammation (D'Ambrosi et al., 2014). In other models of cell senescence, cytoplasmic p16 inhibits the nuclear translocation of phosphorylated regulators affecting stress responses, such as ERK1/2 (also known as MAPK3/1) (Chen et al., 2020). These results might align with previous findings of increased phosphorylated (p)ERK1/2 in ALS samples (Ayala et al., 2011) and would also agree with TDP-43 loss of function in the G93A model, discussed below. Furthermore, recent data show that nuclear TDP-43 is required for repairing DNA double-strand breaks (Mitra et al., 2019), a type of DNA damage associated with cell senescence and ALS (Wald-Altman et al., 2017; Cacabelos et al., 2014). Whether cell senescence is associated causally with disturbances in TDP-43 function will be the focus of future studies.

The potential contribution of cytoplasmic p16 and p21 to cell cycle arrest linked to senescence is unknown. It is known that cytoplasmic p21 (Lee and Helfman, 2004; Koster et al., 2010) and p16 (Evangelou et al., 2004) could interact with mediators of cell cycle. Nuclear P53/p21 and pRB/p16 are the two major pathways – in addition to telomere shortening/dysfunction – for inducing senescence (Serrano et al., 1997). *Terc* loss (i.e. shorter telomere length) advances the phenotype impairment in the G93A mouse model (Linkus et al., 2016). In the same report, the authors indicated that, in ALS patients, microglial cells showed near-to-significant loss of telomere length, which would be in line with our finding of increased p16 levels in Iba1^+^ cells. Regarding the implication of cytosolic p16 in cell cycle blockade, increased cytoplasmic p16 is observed in doxorubicin-induced senescence (Spallarossa et al., 2010), where cytosolic p16 does not change the cell cycle but induces cytoskeletal changes. Our results are also compatible with the cytoskeleton being altered in the G93A mouse model (Maximino et al., 2014) and with specific ALS forms being caused by several mutations in genes that regulate cytoskeleton (Castellanos-Montiel et al., 2020). Our findings suggest that increased p16/p21 in G93A are both cytoplasmic and nuclear. When added to the limited contribution of cytosolic p16/p21 as cell cycle inhibitors, the results reported here shall be better described as an increased expression of senescence biomarkers rather than bona fide senescence itself.

Lee et al. (2006) authors described the origin of SA-β-gal activity, another senescence marker, as being directly linked to lysosomal density. Our data on SA-β-gal activity in neurons suggest that, in contrast to in the aging mouse brain (Piechota et al., 2016), this typical marker is not associated with potential senescence. Motor neurons contain more lysosomes than other cells (Xie et al., 2015a). Of note, lysosome biogenesis (Xie et al., 2015a) and lysosomal density (Xie et al., 2015b) are compromised in this ALS mouse model, potentially explaining our results from the SA-β-gal activity assay.

Another marker commonly employed in senescence description is the cytokine increase linked to SASP. In this case, we quantified the expression of typical SASP markers *Il1a* and *Il6*, and we analyzed by an antibody array the tissue levels of several related inflammatory proteins. *Il1a* mRNA is increased in the presymptomatic stage and is known to be the upstream regulator of IL-6 in SASP (Orjalo et al., 2009). IL-6 is increased in cerebrospinal fluid in ALS, Alzheimer's disease and Parkinson's disease (Chen et al., 2018). Overall, our results might reflect a complex interaction between senescence, SASP, and changes in reactive glial cells and neurodegeneration. Comparison of our data with publicly available RNASeq datasets enhances the robustness of these findings. Both microglia and astrocyte RNASeq data confirm the increased amount of *p21*, which may be implicated, as explained above, in mitotic arrest and in cytoskeletal changes. Furthermore, these results also demonstrated increased *Csf1* mRNA in microglia, in line with our finding of macrophage-colony stimulating factor (M-CSF) (its derived protein) found in G93A mice and with data on ALS patients (Akiyama et al., 1994). Interestingly, administration of GW2580, an antagonist of microglial M-CSF signaling, improves survival in G93A mice (Martínez-Muriana et al., 2016). M-CSF has also been detected in other preclinical models of ALS, including TDP-43-related ones (Hunter et al., 2021). Recent data show that M-CSF may be implicated in cellular senescence of skeletal stem cells (Ambrosi et al., 2021), and this cytokine is implicated in p53-mediated cell arrest (Azzam et al., 2013). The antibody array employed here also revealed the increased amount of TNF receptors, already involved in SASP (Schafer et al., 2020), confirming the potential role of TNF signaling in ALS (reviewed in Hensley et al., 2006) and other neurodegenerative processes (Yuan et al., 2019). Regarding TCA-3, the product of *Ccl1*, no previous implication in ALS has been reported. As TCA-3 is required for the clearance of senescent cells in cancer (Vilgelm et al., 2015), we speculate that its buildup in this model could signal microglial chemotaxis (Li et al., 2020) and that it is not directly related to cell senescence. In contrast, TIMP-1, another of the differential markers shown here, is a soluble factor implicated in the reprogramming of SASP, again in the context of cancer (Guccini et al., 2021), and it has been involved as a neuroprotective response in G93A mice (Izrael et al., 2018). Additionally, sex-induced differences in SASP reported here agree with the fact that there are sex differences in ALS, with men having a higher incidence and prevalence than women and unique clinical characteristics (men deteriorate more quickly) (reviewed in Blasco et al., 2012). These results can also be seen in experimental ALS models (Ajroud-Driss and Siddique, 2015). Additionally, neuroinflammatory conditions varied noticeably between mouse sexes (Sorge et al., 2015). Thus, the sex-induced differences in SASP shown here may be explained by estrogen-sensitive variations in microglia and lymphocyte function (Villa et al., 2018). In addition to its well-known role as an antineuroinflammatory factor (Vegeto et al., 2003), estrogen has protective activity that could be derived from other properties. Recent data in an unrelated preclinical model of neurodegeneration revealed that tamoxifen, a selective estrogen receptor antagonist, can increase lysosomal function and autophagy (Soldati et al., 2021), involving the signaling through the transcription factor EB. Because loss of this transcription factor has been causally linked to ALS (Cunningham et al., 2020), we could speculate that part of the protective effect of feminine sex could be related to this pathway. Other estrogen-dependent mechanisms could underlie their activity, enhancing endolysosomal acidification in neurons (Datta et al., 2021). Similarly, it is known that the activation of selected estrogen receptors, such as GP30, protects neurons by modulating autophagy in an excitotoxicity context (Yue et al., 2019).

The present data are the first to show specific alterations regarding splicing function controlled by TDP-43 in the hSOD1-G93A mouse model of ALS. Notably, loss of splicing correlates with the senescence marker *p16*. The present study is one of the first reports regarding cell senescence, TDP-43 dysfunction and motor neuron disease. TDP-43 pathology is present in nervous tissue during the normal aging process and age-related neurodegenerative disorders, such as Alzheimer's disease and limbic-predominant age-related TDP-43 encephalopathy (Torres et al., 2020). Because G93A seems to accelerate age-related traits, including the buildup of senescence biomarkers, as demonstrated here, we evaluated whether senescence traits were associated with TDP-43 dysfunction. Aging is linked to alterations in the splicing machinery (reviewed in Deschênes and Chabot, 2017), with age-related alteration of expression and activity of splicing regulators, such as SRSF2, promoting a cascade of splicing alterations to affect the maintenance of genomic, chromatin and DNA integrity. It remains controversial whether TDP-43 pathology is present in these mice, with previous studies showing mislocalization and biochemical modification (Shan et al., 2009) and others pointing to no pathology in G93A (Robertson et al., 2007). Our findings support the potential impairment of TDP-43 function in line with progressive neuroinflammatory changes.

Our results in two independent ALS models (G93A and Q331K mice) suggest a novel pathogenic venue, as a surrogate of TDP-43 function, ADIPOR2, is an essential regulator in intracellular lipid composition sensing (Ruiz et al., 2019). ADIPOR2 is a member of the ADIPOR family, evolutionarily conserved regulators of membrane homeostasis that work as membrane fluidity sensors and regulate phospholipid composition. Membrane rigidification triggers ADIPOR signaling, which promotes fatty acid desaturation and polyunsaturated fatty acid incorporation into membrane phospholipids until fluidity is restored (Ruiz et al., 2019). Cellular lipid homeostasis is affected in ALS, as shown by recent analyses in cellular models and tissue samples (Ramírez-Nuñez et al., 2021; Sol et al., 2021). Our data indicate that senescence-associated TDP-43 disturbances could be pathogenic through loss of *Adipor2* mRNA. Whether this holds true for ALS patients will be focus of future research.

Results on Navitoclax treatment reinforce differences in molecular effectors between different neurodegenerative processes such as Alzheimer's disease and ALS. Navitoclax is an inhibitor of the antiapoptotic protein Bcl-2 (Zhu et al., 2016), essential for survival of senescent cells. Senolysis is achieved when this antiapoptotic protein is inhibited, promoting cell death (Zhu et al., 2015). Navitoclax treatment was not enough to slow the disease progression and did not extend the survival in our G93A mice. In contrast to data from Alzheimer's disease and Parkinson's disease models, we observed that Navitoclax does not prevent the increase in senescence and SASP markers. We hypothesize that the senescence-like phenotype in the G93A model is not driven by the *Bcl2* expression of stressed or aged cells (Zhu et al., 2015), supporting previous data (Vukosavic et al., 1999).

Further studies are warranted to determine whether senescence-linked phenomena are mechanistically involved in ALS, clearing the pathway for therapeutic development. As a limitation of our work, we acknowledge the lack of unequivocal mechanistic insight, which may be obtained by crossbreeding G93A mice with the p16-INK4A-specific (Baker et al., 2011) or similar cellular ablation models in the future. We envisage that the translational power of senolytic treatments assayed here would be of use for further development of future therapeutic schemes. Indeed, Bcl-XL, a Bcl-2 family member, is overactive in astrocytes from G93A, providing pro-survival input, and may mediate the activation of toxic astroglia (Lee et al., 2009). Thus, specific inhibition of Bcl-XL could significantly affect disease progression. Results from *in vitro* modeling of cell senescence by fibroblast culture confirm the potential of the dasatinib and quercetin combination in the G93A preclinical model. Further, these results suggest that cell culture (as shown for Navitoclax) might model *in vivo* events, as this Bcl-2/Bcl-XL/Bcl-w inhibitory agent was unable to prevent *in vitro* or *in vivo* changes. Also, these results illustrate the complexity of senescence-like phenotypes impinged by different neurodegeneration-associated noxa.

The LSC from the hSOD1-G93A mouse, a model of familial ALS, exhibits a non-canonical profile of senescence biomarkers. This profile is characterized by an early increase in *p16* and a late increase in *p21*, with both displaying a mainly cytoplasmic pattern in glial cells without an increase in SA-β-gal activity. In the case of SASP, it also has a dynamic profile, with increasing levels of *Il1a* from the presymptomatic stage onward and a sharp peak of expression in end-stage transgenic mice. Regarding AS, this tissue shows a dysfunctional splicing activity of TDP-43 in end-stage ALS mice. This is the first time that senescence markers, SASP and TDP-43-associated splicing dysfunction have been described in this ALS mouse model.

## MATERIALS AND METHODS

### Animal experiments

A colony of the strain B6.Cg-Tg(SOD1*G93A)1Gur/J (JAX stock #004435; referred to as hSOD1-G93A or G93A) was purchased from The Jackson Laboratory (Bar Harbor, MN, USA). Mice were maintained in C57BL/6J background. Genotyping was performed following The Jackson Laboratory's instructions. After genotyping and weaning, animals were placed in a 12:12 h dark/light cycle, at 22±2°C temperature, 50±10% relative humidity, in individual cages (at 21 days). We evaluated three different ages: 90 days (early clinical phase), 120 days and 150 days (defined as end stage, characterized by bilateral hindlimb paralyses) for age-related studies. Navitoclax (T2101, Targetmol) was diluted in 60% Phosal 50 PG (Lipoid), 30% PEG400 (91893, Sigma-Aldrich), 10% EtOH. Navitoclax was administered by oral gavage at a 50 mg/kg body dose for five consecutive days followed by 16 days of rest (*n*=5 per group). Treatment cycles were repeated until the clinical endpoint (righting reflex >20 s), which was achieved at the mean age of 170 days. Spinal cords and sciatic nerves were rapidly excised, frozen in liquid N_2_ and stored at −80°C.

A colony B6.Cg-Tg(Prnp-TARDBP*Q331K)103Dwc/J (JAX stock #017933; referred to as Q331K) was purchased from The Jackson Laboratory. Mice were placed under the same conditions as the G93A colony. Mice were maintained in C57BL/6NJ background. Ten non-transgenic mice and six transgenic littermates were euthanized at 150 days old, and LSCs were rapidly excised, snap-frozen in liquid N_2_ and stored at −80°C. This study was approved by the Animal Research and Ethics Committee at the University of Lleida. The care and use of all experimental animals employed here complied with relevant local animal welfare laws, guidelines and policies.

### Cell culture

3T3 cells, obtained from American Type Culture Collection (#CCL-173, authenticated by provider) were maintained in Dulbecco's modified Eagle medium (DMEM; 11965, Thermo Fisher Scientific), 10% fetal bovine serum (FBS; 10270, Thermo Fisher Scientific), 100 U/ml penicillin–streptomycin (15140-122, Thermo Fisher Scientific) at 37°C and 5% CO_2_. For silencing, 20 nM (final concentration) *Tardbp* siRNA mmsiTDP-43s (5′-AGGAAUCAGCGUGCAUAUA-3′), mmsiTDP-43as (5′-UAUAUGCACGCUGAUUCCU-3′; siTdp-43) or scrambled siRNA (SCR) was mixed in 100 µl Opti-MEM (31985062, Thermo Fisher Scientific) with 2 µl RNAiMAX (13778100, Thermo Fisher Scientific) on the bottom of a well from a six-well plate and incubated for 20 min at room temperature. Then, 2 ml of DMEM (11965092, Thermo Fisher Scientific) supplemented with 10% FBS containing 100,000 3T3 cells/well were seeded onto transfection mix. After 24 h, transfection medium was removed and changed to DMEM+10% FBS. At 48 h post-transfection, cells were collected for posterior analyses.

Fibroblasts were obtained from the ears of G93A and non-transgenic littermates. Briefly, both ears were digested using 0.2 U/ml type II collagenase (LS004174, Worthington Biochemicals) for 45 min at constant shaking at 37°C. Every 15 min, supernatant was collected and centrifuged for fibroblast isolation and to prevent cell damage by excessive digestion. Digestion solution was replaced and incubated for 15 min three times. Fibroblasts were finally seeded in 60 mm plates and cultivated in DMEM+10% FBS. Non-senescent fibroblasts were collected at 10 days in vitro (DIV) and senescent fibroblasts at 20 DIV (when they stopped growing). Cells were then treated with dasatinib (250 nM)+quercetin (50 μM), Navitoclax (20 μM) or vehicle [dimethyl sulfoxide (DMSO)] for 72 h [modified from (Zhu et al., 2016)]. RNA was extracted using the TRIzol–chloroform method, as detailed below.

### Western blotting

Spinal cord lysates were prepared to homogenize on ice (1:20, weight: volume) in radioimmunoprecipitation (RIPA) buffer with Protease Inhibitor Cocktail (1×) using a homogenizer device (T10 basic UltraTurraX, IKA, Staufen, Germany). After sonication, protein quantification was performed with Bradford assay (5000006, Bio-Rad). Fifteen micrograms of protein were loaded onto a 12% acrylamide SDS-PAGE gel. Membranes were blocked with I-Block (T2015, Thermo Fisher Scientific) for 1 h and incubated overnight with primary antibody anti-TDP-43 (10782-2-AP, Proteintech; 1:1000) in Tris-buffered saline containing 0.05% Tween 20 (TBS-T). After primary antibody incubation, membranes were washed three times with TBS-T and incubated with secondary antibody for 1 h. Immobilon™ Western Chemiluminiscent HRP Substrate (WBKLS0500, Merck Millipore) was used for immunodetection. For normalization, membranes were stained with Coomassie Brilliant Blue G (27815, Sigma-Aldrich). Incubation without primary antibody was employed for ensuring specificity of bands, the intensities of which were quantified with ImageLab v5.2.1 (Bio-Rad).

### Antibody array

For further analyses of inflammatory profiles, we employed an antibody array (AAM-INF-1-4, RayBio) in spinal cord lysates from control (*n*=4, two male) and G93A (*n*=4, two female) 100-day-old mice according to manufacturer instructions. Briefly, spinal cord lysates, obtained and quantified as above for western blotting, were incubated with the arrays overnight at 4°C, then incubated with biotinylated secondary antibodies and horseradish peroxidase. The bioluminescence emitted by the arrays was quantified with ImageLab v5.2.1 (Bio-Rad), and dot quantification was performed by measuring the optical density of each dot using the oval tool in ImageJ. The same area was used to quantify all the arrays. The data were analyzed following the manufacturer's instructions.

### RNA extraction, cDNA synthesis and RT-qPCR

First, 1 ml TRIzol reagent (AM9738, Thermo Fisher Scientific) was added to 50-100 mg tissue. The tissue was then mechanically homogenized (T10 basic UltraTurraX). Then, 200 µl chloroform was added to each sample and mixed. After 5 min of incubation at room temperature, the samples were centrifuged at 12,000 ***g*** (15 min, 4°C) to separate the phases. The aqueous phase was transferred into a new tub and mixed by vortexing with 500 µl isopropanol. After incubation for 10 min at room temperature, RNA was precipitated through centrifugation at 12,000 ***g*** (10 min, 4°C). The resulting supernatant was removed, and the pellet was washed with 75% ethanol. After vortexing, the samples were centrifuged again at 12,000 ***g*** (10 min, 4°C). The supernatant was discarded, and the RNA pellet was allowed to air dry at room temperature. The RNA was resuspended with 50 µl RNAse-free water, quantified with an ND-1000 UV/Vis spectrophotometer (Nanodrop Technologies) and stored at −80°C until further use. One microgram of RNA was used for retrotranscription employing TaqMan Reverse Transcription Reagent using random hexamers (N8080234, Thermo Fisher Scientific). RT-qPCR experiments were performed using a CFX96 instrument (Bio-Rad) with SYBR Select Master Mix (4472908, Thermo Fisher Scientific). Each 20 µl of reaction mix contained 4 µl cDNA, 10 µl SYBR Select Master Mix, 0.2 nM forwarding primer and 0.2 nM reverse primer solutions, and 4 µl PCR-grade water. The RT-qPCR run protocol was as follows: 50°C for 2 min and 95°C for 2 min, with 95°C for 15 s and 60°C for 1-min steps repeated for 40 cycles; and a melting curve test from 65°C to 95°C at a 0.1°C/s measuring rate. *n*=3-10 mice per group were used for RT-PCR experiments. Primers employed in these experiments are listed in Table S1. *Actb* expression was used as housekeeping gene to normalize other genes. Specific *Adipor2* cryptic mRNA (primers annealed with cryptic exon) was normalized with *Adipor2* standard transcript (the primers annealed with conserved exons).

### SA-β-gal activity

Briefly, paraformaldehyde-fixed frozen sections were incubated with X-gal solution [20 mg/ml X-gal (Sigma-Aldrich), 5 mM K_3_Fe(CN)_6_, 5 mM K_4_Fe(CN)6 and 2 mM MgCl_2_] in PBS at pH 6.0 overnight at 37°C. All samples were assayed simultaneously. Then, neurons were stained with Green Fluorescent Nissl Stain (N21480, Thermo Fisher Scientific) diluted 1:150 in PBS and incubated for 20 min at room temperature. The slices were then washed three times with PBS for 10 min at room temperature and once with PBS for 2 h. Nuclei were stained with 4′,6-diamidino-2-phenylindole (DAPI; 32670, Sigma-Aldrich). Images of the stained sections were taken using an inverted microscope (IX71S8F-2, Olympus). Eight randomly selected areas of each mouse (*n*=2-3 per group) of the ventral horn of LSC sections were photographed at 20× magnification for visual analysis, performed in a blinded fashion. The whole section of SA-β-Gal-stained slices was photographed at 4× magnification.

### IF

One control and one transgenic LSC were fixed in 4% paraformaldehyde made in PBS overnight at 4°C and cryopreserved in 30% sucrose in PBS for 48 h. The LSC was then cut at a 16 µm depth and seeded onto a gelatin-coated slide. Samples were permeabilized with 0.3% Triton X-100 PBS for 30 min and blocked with 5% bovine serum albumin in PBS for 1 h at room temperature. The primary antibodies anti-p16 (ab54210, Abcam; 1:100) and anti-GFAP (ab7260, Abcam; 1:200) or anti-Iba1 (019-19741, FUJIFILM Wako; 1:200) were diluted in 0.3% Triton X-100 PBS and incubated overnight at 4°C. The slices were washed three times with PBS for 5 min at room temperature, and then incubated with the secondary antibody (diluted 1:800 in PBS), goat anti-mouse Alexa Fluor 555 (A21422, Thermo Fisher Scientific) or goat anti-rabbit Alexa Fluor 488 (A11008, Thermo Fisher Scientific), for 1 h at room temperature in the dark. Sections were finally counterstained with 1 µg/ml DAPI in PBS for 10 min at room temperature and mounted on slides with Fluoromount-G® (0100, Southern Biotech). The same procedure was adopted for evaluating anti-p16 cellular distribution in primary fibroblasts. Samples were imaged using a laser scanning confocal microscope (Olympus FluoView FV10). Individual intensities of GFAP, p16 and Iba1 staining were quantified employing the Artificial Intelligence (AI) Software Platform Biodock 2022 (available from www.biodock.ai). We used the ‘Cell Segmentation’ AI pipeline, employing 25 pixels as the mean nucleus diameter and 25 pixels as the growth parameter. Using these conditions, at least 2500 cells were analyzed for GFAP and Iba1 immunostaining.

### IHC

One control and one transgenic paraformaldehyde-fixed paraffin-embedded tissue slide were dried for 1 h at 65° before deparaffinization, rehydration and epitope retrieval in the Pre-Treatment Module (PT-LINK, Agilent Technologies-DAKO) at 95°C for 20 min in 50× Tris/EDTA buffer, pH 9. For p21 immunohistochemical staining, p21WAF1/Cip1 antibody (clone SX118, Agilent Technologies-DAKO; 1:100) was used. After incubation, the reaction was visualized with an EnVision™ FLEX Detection Kit (Agilent Technologies-DAKO) using diaminobenzidine chromogen as a substrate. For p16 immunohistochemical staining, the anti-p16-INK4A antibody was used using a CINtec® Histology Kit (clone E6H4, Roche) following the manufacturer's instructions. Sections were counterstained with Hematoxylin and quantified in a blinded fashion.

### Statistical analysis

All statistical tests and graphs were performed using Prism 6 (GraphPad Software). *P*<0.05 was considered significant. Normalized mRNA expression was analyzed with ordinary two-way ANOVA of the variable time and genotype. Bonferroni's or Fisher’s least significant difference multiple comparisons test was used for multiple comparisons between genotypes. A linear regression was tested to evaluate the relationship between *p16* and *Adipor2* cryptic mRNA levels. Venn diagrams were produced using the online Venny package (v 2.1) (https://bioinfogp.cnb.csic.es/tools/venny/index.html).

## Supplementary Material

Supplementary information
