# Supplementary material for: A motor neuron disease mouse model reveals a non-canonical profile of senescence biomarkers
Source: Dis Model Mech. 2022 Aug 29;15(8):dmm049059. doi: 10.1242/dmm.049059 (PMC9459393; doi:10.1242/dmm.049059)
Supplement: Supplementary information [file dmm-15-049059-s1.pdf]

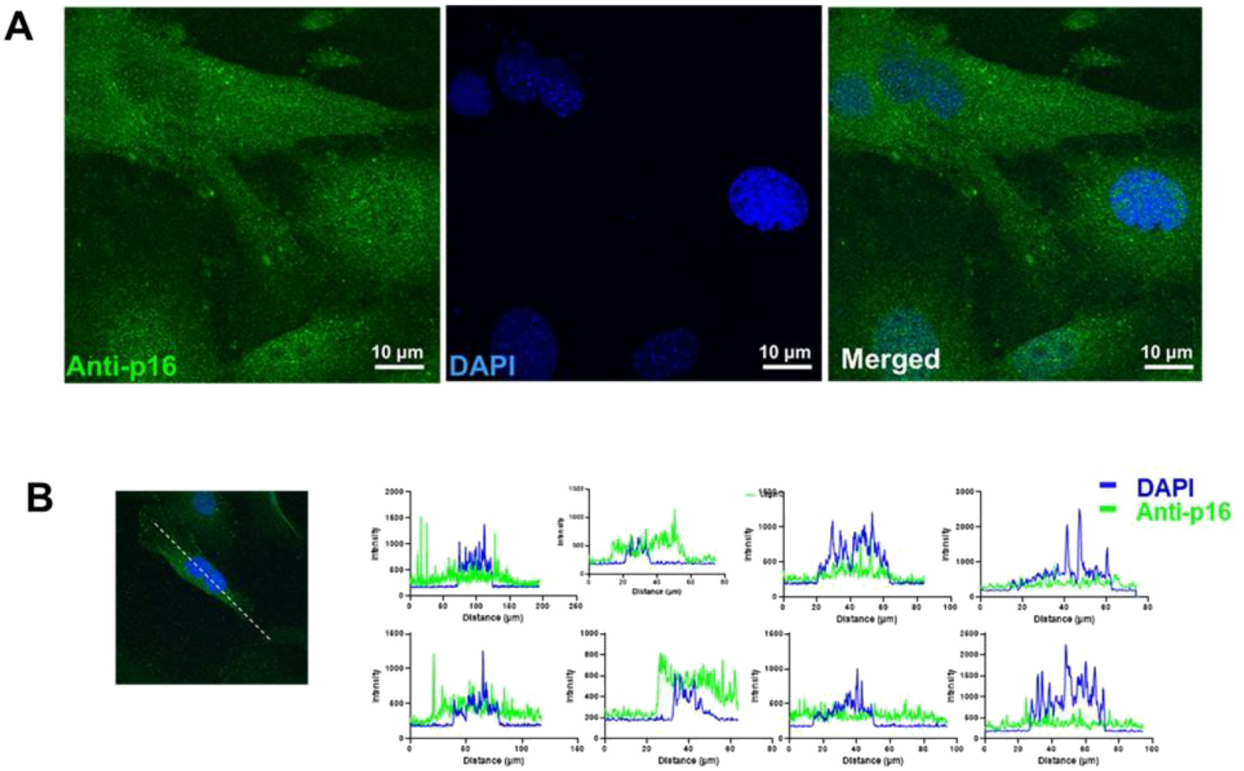

Fig. S1. Cytosolic abundance of anti-p16 immunostaining. A) Representative confocal images showing both cytosolic and nuclear immunoreactivity against anti-p16 in adult mice fibroblasts. B) Abundance profiles of cellular immunoreactivity of anti-p16 (green) in comparison with DAPI fluorescence (blue). The left panel shows a representative cell with the line employed for quantification (white discontinuous).

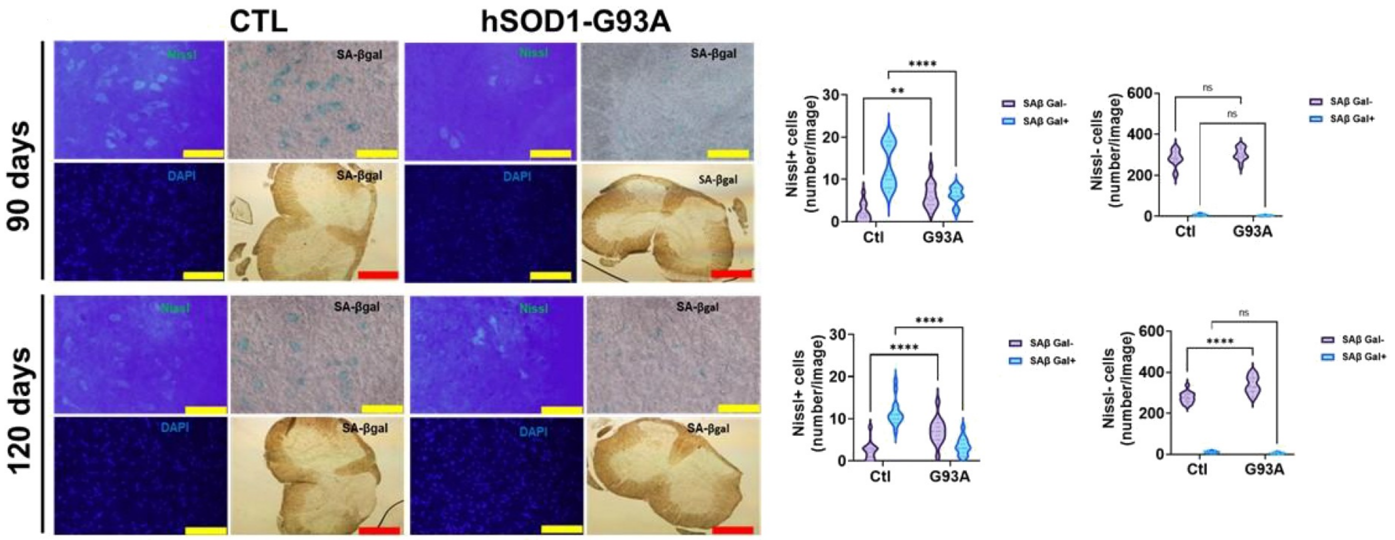

Fig. S2. SA-beta-gal activity was decreased in 90- and 120-day-old hSOD1-G93A mice. Nissl+ cells are the main contributors of this activity, which is lower in ALS mice. In contrast, Nissl- cells exhibited a slight decrease in transgenic mice. ***p < 0.001; ****p < 0.0001. Yellow scale bar represents 500 µm and red scale bar represents 2500 µm.

Disease Models & Mechanisms • Supplementary information

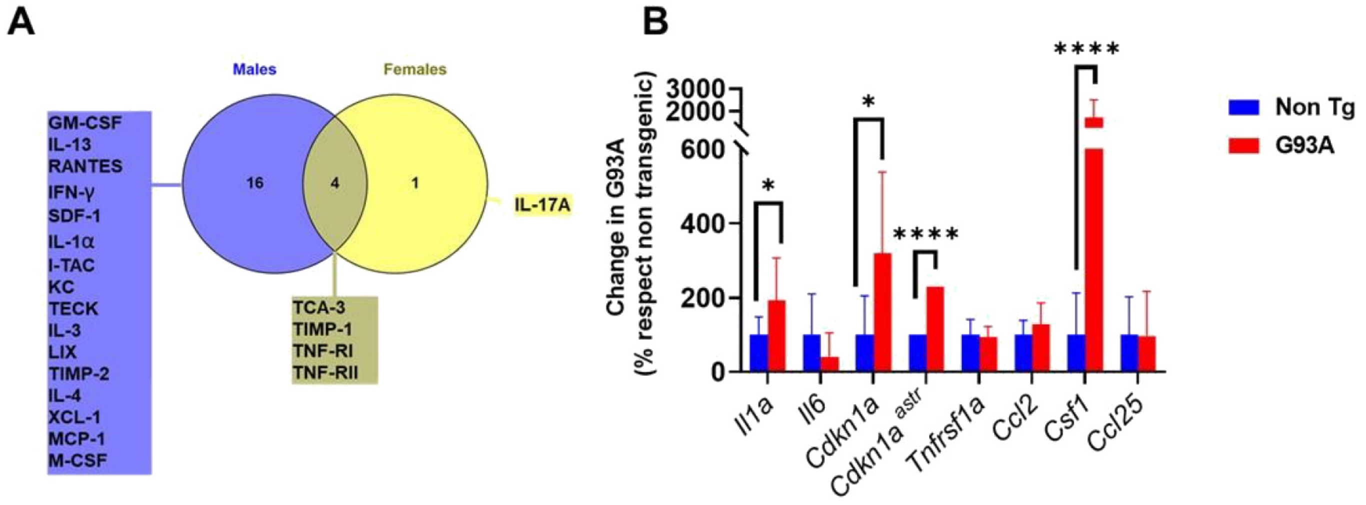

Fig. S3. Sex and cell subtype influences senescence-related phenomena in G93A mice. A) Venn diagram showing the LSC protein significantly affected by G93A expression according the antibody array. B) mRNA levels of selected genes, evaluated by RNAseq in microglial cells (Chiu et al., 2013) and astrocytes (Liu et al., 2020, *Cdkn1a*^{astr}) from G93A mice. Data were obtained from publically available databases, and expressed as mean ±SEM. ns $p > 0.05$; *$p < 0.05$, and ****$p < 0.0001$ by Students t test.

Disease Models & Mechanisms • Supplementary information

Table S1. SYBR Green probes used in RT-qPCR analysis

Gene	Sequence
Actb Fwd	GTGACGTTGACATCCGTAAAGA
Actb Rev	GCCGGACTCATCGTACTCC
p16 Fwd	CCCAACGCCCCGAACT
p16 Rev	GCAGAAGAGCTGCTACGTGAA
Il1a Fwd	AGCGCTCAAGGAGAAGACC
Il1a Rev	CCAGAAGAAAATGAGGTCGG
Il6 Fwd	ACCAGAGGAAATTTTCAATAGGC
Il6 Rev	TGATGCACTTGCAGAAAACA
Ifna Fwd	ATGGCTAGGCTCAGCACTTTC
Ifna Rev	CTCACTCAGACTTGCCAGCA
Ifnb Fwd	AGCTCCAAGAAAGGACGAACA
Ifnb Rev	GCCCTGTAGGTGAGGTTGAT
Adipor2 Fwd	TGTTTGTAAGGTGTGGGAAGG
Adipor2 Rev	GTTGCCCGTCTCTGTGTGTAT
Adipor2 cryptic Fwd	AGAAGTGGAGTTACAATTGTG
Adipor2 cryptic Rev	AAACAAACTCTTCCATTCGTT
p21 Fwd	TTGCCAGCAGAATAAAAGGTG
p21 Rev	TTTGCTCCTGTGCGGAAC

Disease Models & Mechanisms • Supplementary information
